# Chlorpromazine induces hyposalivation by inhibiting muscarinic Ca^2+^ signaling in salivary glands

**DOI:** 10.1007/s00210-025-04438-8

**Published:** 2025-07-23

**Authors:** Yoon-Jung Kim, Yoo-Bin Kim, Soohyun Kim, Tae-Yong Choi, Hee-Kyung Park, Se-Young Choi

**Affiliations:** 1 Department of Physiology, Dental Research Institute, Seoul National University School of Dentistry, Seoul, 03080 Republic of Korea; 2 Department of Oral Medicine and Oral Diagnosis, Dental Research Institute, Seoul National University School of Dentistry, Seoul, 03080 Republic of Korea; 3 Department of Oral Medicine, Seoul National University Dental Hospital, Seoul, 03080 Republic of Korea

**Keywords:** Chlorpromazine, Salivary gland, Hyposalivation, Intracellular Ca^2+^, G-protein–coupled receptor, Store-operated Ca^2+^ entry

## Abstract

Chlorpromazine (CPZ), a dopamine D2 receptor antagonist, is an antipsychotic drug widely used in the treatment of schizophrenia. While CPZ is known to induce hyposalivation, the exact mechanism by which it inhibits secretory function in the salivary glands remains unclear. In this study, we investigated the molecular mechanism underlying CPZ-induced hyposalivation. Our findings confirmed that CPZ decreases salivation by inhibiting muscarinic signaling in a dose-dependent manner in a mouse model. By analyzing a single-cell RNA sequencing (scRNA-seq) database, we searched known CPZ targets in salivary gland cells and found that dopamine receptors are not expressed in these cells. CPZ inhibited the increase in intracellular Ca^2+^ triggered by muscarinic and histamine receptors in human salivary gland cells. This inhibition involved both the release of Ca^2+^ from the endoplasmic reticulum (ER) and the influx of extracellular Ca^2+^ through store-operated Ca^2+^ entry (SOCE). These findings suggest that in salivary glands, CPZ induces hyposalivation by inhibiting Ca^2+^ signaling through multiple sites of action, including muscarinic receptor and SOCE.

**Supplementary Information:**

The online version contains supplementary material available at 10.1007/s00210-025-04438-8.

## Introduction

Many neurologic and psychiatric drugs are known to cause hyposalivation (Cockburn et al. 2017). Saliva plays a crucial role in oral health by hydrating the oral cavity, maintaining humidity, lubricating mastication, buffering oral fluids, and preventing dental caries. Consequently, issues with salivation can lead to various oral diseases (Pedersen et al. 2018). One significant cause of hyposalivation is the use of outpatient prescription drugs (Proctor 2016; Bhattarai et al. 2018). Reports indicate that various drugs, including antihistamines, anticholinergics, and diuretics, can reduce salivation (Furness et al. 2011; Villa et al. 2016; Wolff et al. 2017). A notable class of drugs associated with severe hyposalivation is antipsychotics, commonly prescribed for the treatment of schizophrenia, a psychiatric disorder characterized by both positive symptoms (e.g., hallucinations) and negative symptoms (e.g., abnormal thinking) (Insel 2010). The hyposalivation caused by psychiatric drugs is particularly concerning compared to short-term medications, as these drugs are often administered over extended periods, resulting in prolonged hyposalivation and subsequent oral diseases such as rampant caries.

Chlorpromazine (CPZ) is an antipsychotic drug known for causing severe hyposalivation. Widely used since the 1950 s to treat psychosis and manic syndrome, CPZ is prescribed to alleviate symptoms such as hallucinations and delusions (Conley et al. 1998; Rosenbloom 2002). CPZ effectively alleviates schizophrenia symptoms and reduces relapse rates over six months to two years, earning recognition from the World Health Organization as an essential treatment (Adams et al. 2005). CPZ is well-tolerated at doses below 800 mg per day, but higher doses increase the risk of adverse effects and dropout rates (Dudley et al. 2017). Common side effects include sedation, dizziness, dry mouth, weight gain, orthostatic hypotension, and movement disorders such as Parkinsonism and acute dystonia (Adams et al. 2014). Additionally, the widespread prescription of CPZ, its low cost, and extensive pharmacological data make it an attractive candidate for drug repositioning, including potential use in COVID-19 treatment (Glebov 2020; Nobile et al. 2020; Plaze et al. 2020; Stip et al. 2020; Fred et al. 2021). Like other neuroleptic drugs, CPZ primarily acts as a dopamine D2 receptor antagonist in the central nervous system (Kapur and Mamo 2003; Carpenter and Koenig 2008). However, CPZ also exhibits anti-serotonergic, anti-histaminergic, anti-adrenergic, and anti-acetylcholinergic properties (Kusumi et al. 2015). The receptors for these neurotransmitters and neuromodulators are expressed not only in the nervous system but throughout the body. This complexity poses a significant challenge in elucidating the mechanisms underlying CPZ-induced hyposalivation, complicating efforts to avoid or mitigate its side effects. The exact molecular mechanism by which CPZ inhibits secretory function in the salivary glands remains unknown.

The most important secretagogue in the salivary glands is acetylcholine, secreted from parasympathetic nerves. Impairment of G protein-coupled receptor (GPCR)-mediated Ca^2+^ signaling has been shown to cause salivary dysfunction (Jin et al. 2012). An increase in intracellular Ca^2+^ levels ([Ca^2+^]_i_) ultimately leads to the movement of Cl^−^ to the luminal side of salivary glands via Ca^2+^-activated ion transporters and the translocation of aquaporin-5, a water channel in salivary gland cells, to the lumen, resulting in saliva secretion. Therefore, investigating the effect of CPZ on GPCR-mediated Ca^2+^ signaling regulation in the salivary glands is essential. A series of GPCR functions, including M3R, P2Y2R, histamine H1 receptor, and GPR39 in human salivary glands, have been identified (Baum 1993; Turner et al. 1999; Kim et al. 2009, 2019). Could CPZ-induced hyposalivation similarly be mediated through D2 or other dopamine receptors expressed in salivary gland cells? If so, these receptors would need to be expressed in these cells. However, dopamine receptors have not yet been identified in salivary gland cells. Thus, it is worthwhile to identify the target of CPZ within salivary gland cells.

In this study, we investigated the target of CPZ in human salivary glands. We investigated whether CPZ suppresses carbachol-induced salivation in a mouse model. Additionally, we explored the presence of dopamine receptors in mouse salivary gland cells through transcriptomic analysis. We also examined the effects of CPZ on key components of Ca^2^⁺ signaling in salivary gland cells. This study aimed to identify the molecular target underlying CPZ-induced hyposalivation in mouse salivary glands.

## Materials and methods

### Materials

Carbachol (CCh) and histamine (HA) were purchased from Sigma (St. Louis, MO, USA). CPZ, 2-aminoethyl diphenyl borate (2-APB), 1-(5-chloronaphthalenesulfonyl) homopiperazine hydrochloride (ML-9), GdCl_3_, and 1-[2-(4-methoxyphenyl)−2-[3-(4-methoxyphenyl)propoxy]ethyl-1H-imidazole hydrochloride (SK&F96365) were obtained from Tocris (Bristol, UK). Thapsigargin was purchased from Alomone Labs (Jerusalem, Israel). fura-2/acetoxymethylester (fura-2/AM) was obtained from Molecular Probes (Eugene, OR, USA). Fetal bovine serum, Dulbecco’s Modified Eagle’s Medium (DMEM), and penicillin–streptomycin were purchased from Gibco (Grand Island, NY, USA).

### Cell culture

Human submandibular gland cell line HSG cells were maintained in DMEM supplemented with 10% heat-inactivated fetal bovine serum and 1% penicillin–streptomycin. The cells were cultured in a humidified atmosphere containing 95% air and 5% CO_2_. The culture medium was changed daily, and the cells were subcultured every 3 days.

### Measurement of saliva flow rates

Salivation from mice was measured according to a previously published methods (Bagavant et al. 2018). We collected total saliva from an anesthetized 8-week-old C57BL/6 male mouse. Individual mice were weighed and administered intraperitoneal (i.p.) injections of the indicated CPZ concentrations (0.1 mg/kg to 10 mg/kg) along with 300 µg/kg pilocarpine. Saliva was collected from the oral cavity of individual mice using inert polymer swabs for 15 min, starting 1 min after the pilocarpine injection. The weight of each saliva sample was then measured. The drug preparation for treatment and the associated experiment were typically carried out by different scientists for blinding.

### Measurement of intracellular Ca^2+^ concentrations ([Ca^2^^+^]_i_)

Fura-2 was used to determine [Ca^2+^]_i_ level, as previously reported (Kim et al. 2019). Briefly, cell suspensions were incubated in Locke’s solution (in mM: 154 NaCl, 5.6 KCl, 3.6 NaHCO_3_, 5.6 glucose, 2.2 CaCl_2_, 1.2 MgCl_2_, and 5 HEPES buffer adjusted to pH 7.4) supplemented with 3 μM fura-2/AM for 50 min at 37 °C. We monitored the fluorescence ratios using 340- and 380-nm dual excitation wavelengths. The ratio of resultant intensities was detected at a 500-nm emission wavelength. In order to separate Ca^2+^ release from Ca^2+^ store and Ca^2+^ influx through store-operated Ca^2+^ entry, CCh was treated in extracellular Ca^2+^ -free Locke’s solution (in mM: 154 NaCl, 5.6 KCl, 3.6 NaHCO_3_, 5.6 glucose, 1.2 MgCl_2_, and 5 HEPES buffer adjusted to pH 7), and then Ca^2+^ was subsequently added.

### Analysis of scRNA-seq data

Two published single-cell RNA sequencing (scRNA-seq) datasets of submandibular glands (SMGs) from mice (GEO: GSE175649) (Horeth et al. 2021) and human (GEO: GSE199209) (Horeth et al. 2023) were analyzed using the Seurat 4.1.0 R package. Cells with a total number of molecules (nCount_RNA) less than 40,000, a number of detected genes (nFeature_RNA) between 200 and 5,000, and mitochondrial transcripts less than 50% were included for subsequent analysis, following normalization using Seurat’s logNormalize with a scale factor of 10,000. Dimensional reduction was performed using t-distributed stochastic neighbor embedding (tSNE) or Uniform Manifold Approximation and Projection (UMAP) algorithms on 2,000 highly variable genes, and clustering was carried out using a shared nearest neighbor (SNN) modularity optimization-based clustering algorithm. Clusters were annotated based on the expression of known cell type-specific markers. Epithelial populations were identified by the expression of *Epcam* (all epithelium), *Cftr* (striated duct), *Ngf* and *Egf* (GCT), *Krt14*, and *Krt5* (basal duct), *Gstt1* (intercalated duct), *Acta2* (myoepithelial cells), and *Aqp5* (acinar cells). Non-epithelial clusters were identified by the expression of *Colla1* (stromal cells), *Pecam1* (endothelial), *Cd68* (macrophages), *Icos* (T cells), *Kit* (mast cells), *Nkg7* and *Gzma* (NK cells), *Acta2*^+^*Epcam*^*−*^ (smooth muscle), and *Alas2*^+^ (erythroid). Human SMG populations were identified by the expression of *TP63*, *KRT5*, *KRT14*, *CNN1*, and *ACTA2* (basal and myoepithelial cells), *KRT7/19*, *SLC5A5*, and *MUCL1* (ductal), *PIP*, *LPO*, *STATH*, *MUC5B*, and *TFF3* (acinar), *CD79A*, *CD19*, *IGHM*, and *MZB1* (B cells), *CD3G*, *CD3D*, and *CD3E* (T cells), *AIF1*, *CD68*, and *ITGAX* (monocytes).

### Statistical analysis

Data analysis was conducted using SPSS version 23 software (IBM, Armonk, NY, USA). All quantitative data are presented as the mean ± standard error of the mean (SEM). Statistical analysis was performed using one-way analysis of variance (ANOVA), followed by Bonferroni’s test for post hoc analysis of multiple comparisons.

## Results

### CPZ decreases muscarinic receptor–induced saliva secretion

**Fig. 1:**
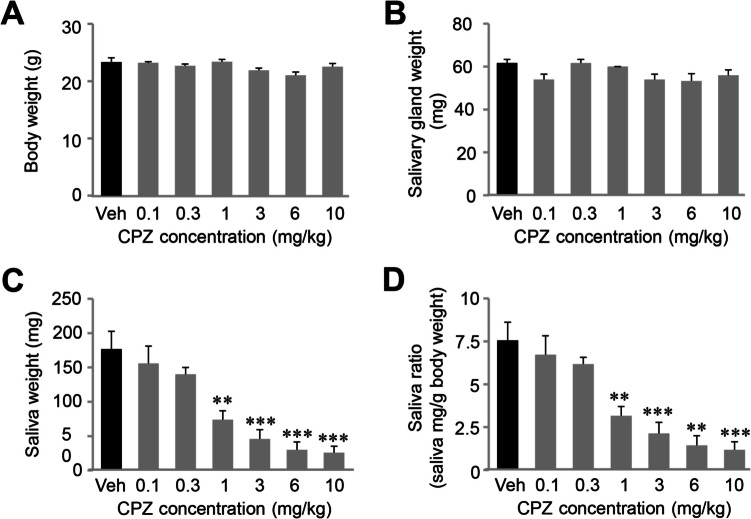
Chlorpromazine (CPZ) reduces muscarinic-induced salivation. Eight-week-old male C57BL/6 mice were anesthetized and administered varying doses of CPZ, followed by measurement of saliva production over 15 min. (A-D) Following intraperitoneal (i.p.) injection of 0.3 mg/kg pilocarpine to stimulate salivation, different doses of CPZ were administered. Body weights (**A**), salivary gland weights (**B**), and salivary output (**C**) were recorded. The saliva ratio (**D**) was normalized to saliva weight per body weight. Data represent mean ± SEM. Vehicle, *n* = 6; CPZ 0.1 mg/kg, *n* = 5; CPZ 0.3 mg/kg, *n* = 3; CPZ 1 mg/kg, *n* = 5; CPZ 3 mg/kg, *n* = 5; CPZ 6 mg/kg, *n* = 3; CPZ 10 mg/kg, *n* = 5; **p < 0.01; ***p < 0.001 compared to vehicle control; one-way ANOVA with Bonferroni post hoc analysis

Although CPZ administration in human patients causes hyposalivation, this xerogenic effect has not been confirmed in a mouse model. We investigated whether CPZ inhibits muscarinic signaling-mediated salivation using a mouse model. We utilized a dosage range of 0.1–10 mg/kg, which aligns with those reported in previous studies. Intraperitoneally administered CPZ has been demonstrated to modulate TNF production (4 mg/kg) (Mengozzi et al. 1994), alter doxorubicin concentrations (15 mg/kg) (Lundgren-Eriksson et al. 1997), and alleviate muscle cramps and motor stereotypy (10 mg/kg) (Andreev-Andrievskiy et al. 2017). Varying doses of CPZ were administered intraperitoneally, and salivary secretion was stimulated with pilocarpine (300 µg/kg i.p. injection), a muscarinic receptor agonist. This resulted in a dose-dependent decrease in the weight of secreted saliva with an IC_50_ of 0.906 ± 0.487 mg/kg (Fig. 1C) without changes in either body weight or salivary gland weight (Fig. 1A-D). These findings confirm that CPZ induces hyposalivation by inhibiting muscarinic signaling in a mouse model.

### Searching for molecular targets of CPZ-induced hyposalivation through reanalysis of scRNA-seq datasets

**Fig. 2:**
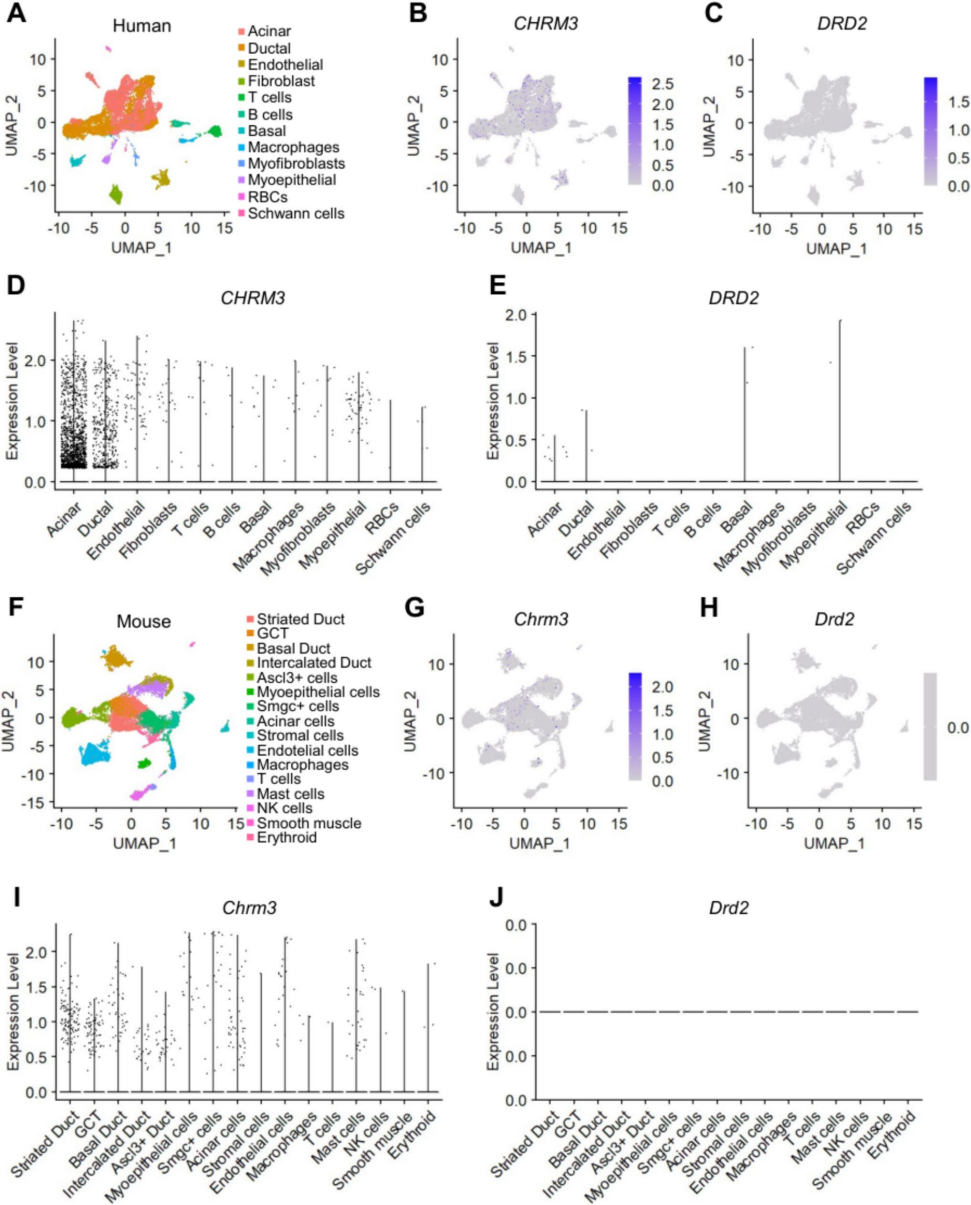
Single-cell RNA sequencing (scRNA-seq) analysis of cell types and mRNA expression in human and mouse submandibular glands (SMGs). (**A**) UMAP plot of human SMGs. scRNA-seq dataset available at GEO: GSE199209. (**B**, **C**) UMAP plots showing *CHRM3* and *DRD2* gene expression in human SMGs. (**D**, **E**) Violin plots displaying *CHRM3* and *DRD2* gene expression in human SMGs. (**F**) UMAP plot of mouse SMGs. scRNA-seq dataset available at GEO: GSE175649. (**G**, **H**) UMAP plots showing *Chrm3* and *Drd2* gene expression in mouse SMGs. (**I**, **J**) Violin plots displaying *Chrm3* and *Drd2* gene expression in mouse SMGs

To identify the molecular targets of CPZ-induced hyposalivation, we searched for potential targets in the salivary glands previously reported as functional targets of CPZ. We reanalyzed publicly available single-cell transcriptomic datasets of submandibular gland tissues in both mice (Horeth et al. 2021) and humans (Horeth et al. 2023). Notably, *Drd2* or *DRD2* (Fig. 2A) and other dopaminergic receptors (Figures S1, S2) were not expressed in the SMGs of either species.

In contrast, *Chrm3* or *CHRM3* was expressed in various cell types within the SMGs of both mice and humans (Fig. 2B). Based on these findings, we tested the [Ca^2+^]_i_ increase evoked by dopamine and carbachol. We observed that dopamine did not induce a [Ca^2+^]_i_ increase in HSG cells, while carbachol and histamine did (Figure S3). This suggests that muscarinic receptors and subsequent Ca^2+^ signaling, rather than dopaminergic receptors, may be involved in CPZ-induced reduction of salivation.

### CPZ inhibits muscarinic and histamine receptor–induced [Ca^2+^]_i_ increases in Human Salivary Gland HSG cells

**Fig. 3:**
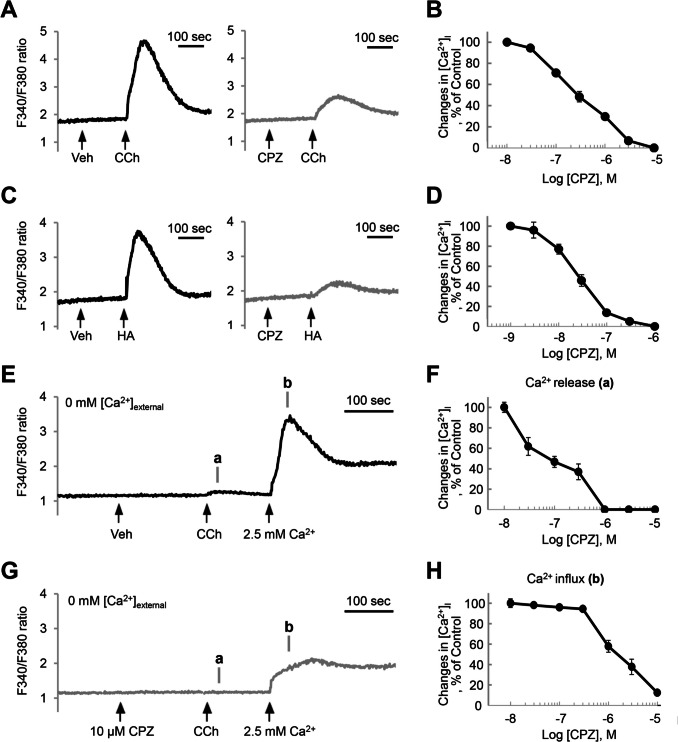
CPZ inhibits muscarinic-induced intracellular Ca^2+^ ([Ca^2+^]_i_) increase via both release and Ca^2+^ influx pathways. (**A**, **C**) Fura-2–loaded HSG cells were preincubated with vehicle (black) or 1 µM CPZ (light gray), then stimulated with 300 μM carbachol (CCh, A) or 100 μM histamine (HA, C). The change in fluorescence ratio (F340/F380) was monitored. (**B**, **D**) Cells were preincubated with CPZ at indicated concentrations, and the peak [Ca^2+^]_i_ levels were quantified after stimulation with 300 μM CCh (B) or 100 μM histamine (D). (**E**, **G**) Cells in Ca^2+^-free conditions were preincubated with vehicle (E) or 10 µM CPZ (G), then stimulated with 300 μM CCh and subsequently restored to 2.5 mM extracellular Ca^2+^. The change in fluorescence ratio (F340/F380) was monitored. (**F**, **H**) In Ca^2+^-free conditions, cells were preincubated with CPZ at indicated concentrations, and CCh-induced [Ca^2+^]_i_ release (F) and influx (H) were quantified at designated time points (**a** and **b**)

We determined whether CPZ affects intracellular Ca^2+^ signaling triggered by muscarinic receptor activation in HSG cells. In cellular studies, CPZ is commonly applied at concentrations ranging from 1–30 μM, including its effects on L-type voltage-sensitive Ca^2+^ channels (1–100 μM) (Ito et al. 1996), IL-1β secretion (20 μM) (Labuzek et al. 2005), store-operated Ca^2+^ entry (SOCE) (30 μM) (Choi et al. 2001), hERG channels (10–100 μM) (Thomas et al. 2003; Lee et al. 2004), Kv1.3 channels in rat megakaryocytes (100 μM) (Kazama et al. 2015), and Kv1.3 channels in medial prefrontal cortex microglia (30 μM) (Lee et al. 2025). Pretreatment with CPZ inhibited the CCh-induced [Ca^2+^]_i_ increase (Fig. 3A) in a concentration-dependent manner, with an IC_50_ of 489 ± 440 nM (Fig. 3B). These inhibitory effects were also detected on the histamine receptor, another Ca^2+^-mobilizing GPCR in HSG cells, with an IC_50_ of 36 ± 14 nM (Fig. 3C, D). This suggests that CPZ inhibited the [Ca^2+^]_i_ increase evoked by the muscarinic and histamine receptors, the predominant GPCRs in salivary gland cells. Interestingly, we found that the CCh-induced [Ca^2+^]_i_ increase in an extracellular Ca^2+^-free condition was more sensitive to CPZ (Fig. 3E-3H). The IC_50_ of CPZ for inhibiting CCh-induced Ca^2+^ release was determined to be 229 ± 119 nM (Fig. 3F), while its IC_50_ for inhibiting CCh-induced Ca^2+^ influx was notably higher at 2.4 ± 2.3 μM (Fig. 3H). This indicates that CPZ differentially inhibits Ca^2+^ release and Ca^2+^ influx following GPCR activation, suggesting that CPZ inhibits not only GPCRs but also SOCE.

### HSG cells have Gd^3+^-resistant SOCE that is inhibited by CPZ

**Fig. 4:**
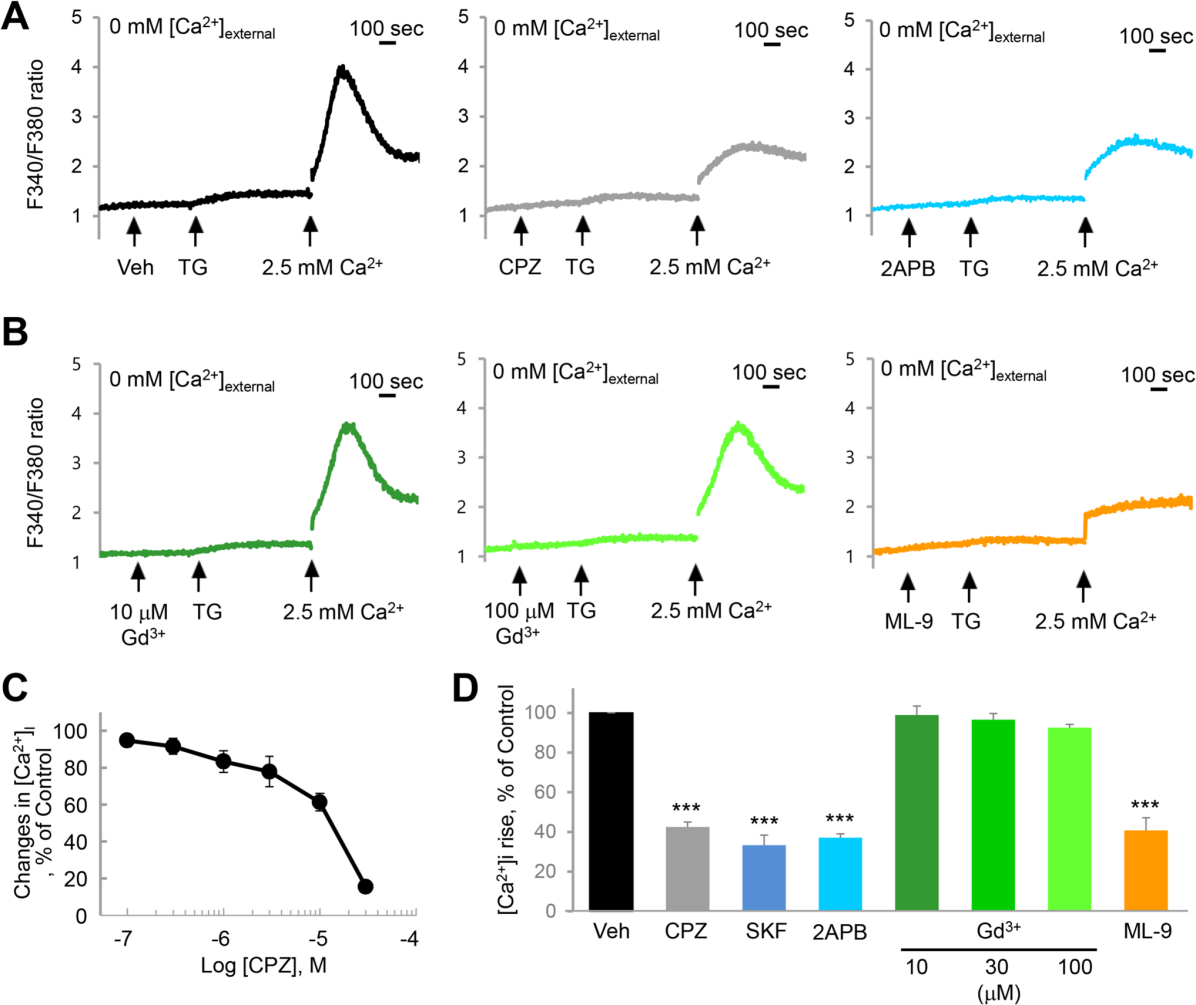
CPZ inhibits thapsigargin (TG)-induced store-operated Ca^2+^ entry (SOCE). (**A**-**B**) Fura-2–loaded HSG cells pretreated with vehicle (black), 30 µM CPZ (gray), 20 µM 2-APB (sky blue), 10 µM Gd^3+^ (green), 100 µM Gd^3+^ (light green), or 100 µM ML-9 (orange) in Ca^2+^-free conditions were stimulated with 1 μM TG, followed by 2.5 mM CaCl_2_ addition to induce Ca^2+^ influx. The change in fluorescence ratio (F340/F380) at [Ca^2+^]_i_ level was monitored. (**C**) Cells were preincubated with CPZ with the indicated concentrations, and the TG-induced peak [Ca^2+^]_i_ was quantified. (**D**) Peak levels of TG-induced [Ca^2+^]_i_ influx were quantitatively analyzed and expressed as a percentage of the vehicle control. Vehicle (Veh), *n* = 3; 10 μM CPZ, *n* = 3; 20 µM SK&F96365 (SKF), *n* = 5; 20 μM 2-APB, *n* = 4; 10 μM Gd^3+^, *n* = 3; 30 µM Gd^3+^, *n* = 3; 100 µM Gd^3+^, *n* = 3; 100 µM ML-9, *n* = 3; ***p < 0.001 compared to vehicle control; one-way ANOVA with Bonferroni post hoc analysis

We confirmed whether CPZ inhibits SOCE in HSG cells. After CPZ pretreatment in an extracellular Ca^2+^-free condition, 1 μM of thapsigargin, a sarcoplasmic/endoplasmic reticulum Ca^2+^ ATPase (SERCA) inhibitor, was added, followed by the restoration of normal extracellular Ca^2+^ concentration (2.5 mM) to induce SOCE by adding CaCl_2_. CPZ decreased the SOCE-mediated [Ca^2+^]_i_ increase in a concentration-dependent manner with an IC_50_ of 16.5 ± 6.3 μM (Fig. 4A, C). We further analyzed the characteristics of SOCE in HSG cells using SK&F96365 (TRPC and STIM1 inhibitor) (Parekh 2010), 2-APB (IP_3_ receptor antagonist) (Xu et al. 2016), ML-9 (STIM1 inhibitor) (Salmon and Ahluwalia 2010), and Gd^3+^ (Orai channel inhibitor) (Chauvet et al. 2016). Interestingly, the TG-induced [Ca^2+^]_i_ increase in HSG cells was inhibited by 2-APB, SK&F96365, and ML-9, but no inhibitory effect was observed with Gd^3+^ (Fig. 4A, B). We also found that Gd^3+^, known to block Orai channels at nanomolar-micromolar concentrations, failed to inhibit SOCE in HSG cells even at significantly higher doses (100 μM), (Fig. 4D). These results suggest that SOCE in HSG cells is distinct from typical Orai-dependent SOCE, but CPZ inhibits Gd^3+^-resistant SOCE in HSG cells, contributing to CPZ’s hyposalivation effect.

## Discussion

Patients undergoing prolonged treatment with medications that cause hyposalivation are at an increased risk of developing secondary oral diseases. Therefore, understanding the xerogenic mechanism of antipsychotic drugs, such as CPZ, is crucial, particularly given the long-term pharmacotherapy required for schizophrenia, which can extend over several months.

This study investigates the molecular target of CPZ in the salivary glands. One main reason the mechanism of CPZ-induced hyposalivation remains unclear might be that CPZ is a well-known antagonist of the D2 receptor. However, no dopamine receptors have been identified in salivary gland cells to date. Therefore, the first step is to verify the presence of dopamine receptors in these glands. After confirming CPZ-mediated hyposalivation in mice (Fig. 1), we searched the publicly available scRNA-seq database and found that none of the dopamine receptors were expressed in the salivary gland cells (Fig. 2). This is not surprising, as many other drugs with less well-defined mechanisms in the salivary glands are also associated with hyposalivation as an off-target side effect (Guggenheimer and Moore 2003). However, trials to find and identify the targets of these drugs in the salivary glands are valuable.

One well-understood mechanism of hyposalivation is the inhibition of muscarinic Ca^2+^ signaling. Medications such as atropine for acute symptomatic bradycardia, scopolamine for motion sickness, and pirenzepine for peptic ulcer disease cause hyposalivation by inhibiting the muscarinic receptor, the primary molecular target of salivation (Carmine and Brogden 1985; Galili et al. 2019). Our transcriptomics analysis clearly shows the expression of M3 receptors in salivary gland cells (Fig. 2). Since our results and previous reports commonly show that CPZ inhibits M3 receptors, M3 could be a potential target of CPZ (Fig. 3). In salivary gland cells, muscarinic receptors are typical activators of phospholipase C (PLC)-β–linked Ca^2+^ signaling. Antihistamines suppress salivation by inhibiting the histamine H1 receptor, which also triggers PLC-β–linked Ca^2+^ signaling in salivary gland cells (Kim et al. 2009). We also show that another target of CPZ could be H1 receptors in salivary gland cells (Fig. 3). The effect of CPZ on the PLC-β downstream pathway is worth investigating because CPZ is known to directly inhibit bradykinin-mediated noradrenaline release by inhibiting SOCE in PC12 cells (Choi et al. 2001). The [Ca^2+^]_i_ elevation through muscarinic receptor and PLC-β activation is consisted by Ca^2+^ release from the ER and Ca^2+^ influx via SOCE. Our results demonstrate that CPZ inhibits both the release and influx steps (Fig. 3), suggesting that CPZ acts at multiple inhibitory sites in the Ca^2+^ signaling pathways. Specifically, CPZ at typical therapeutic concentrations primarily modulates HA and CCh activity, whereas high-dose exposure may induce synergistic hyposalivation via SOCE. This observation enhances the significance of our study, as it aids in interpreting the dose-dependent regulation of salivary gland function by CPZ.

Our study is the first to propose a mechanism for CPZ-induced hyposalivation by revealing its inhibition of Ca^2+^ release and SOCE in salivary gland cells.

Our results strongly indicate that SOCE plays a significant role in salivation, providing further insight into how drugs that promote hyposalivation exert their effects. Thus, we attempted to systematically characterize the SOCE in salivary gland cells. SOCE is mainly modulated by STIM1 and Orai1 (Yuan et al. 2009; Hogan et al. 2010). STIM1, located in the ER membrane, acts as a Ca^2+^ sensor, undergoing a conformational change when ER Ca^2+^ concentration decreases. This change allows STIM1 to form a Ca^2+^ entry channel with Orai1 or TRPC channels present in the plasma membrane (Yuan et al. 2009; Hogan et al. 2010; Cheng et al. 2013). Numerous studies have shown that the properties of SOCE vary depending on the cell type. For instance, SOCE in T cells is completely inhibited by 10 μM Gd^3+^, which is characteristic of Orai1-dependent SOCE (Lee et al. 2017). However, SOCE in PC12 cells exhibits resistance to Gd^3+^, even at concentrations as high as 100 μM, far exceeding the nanomolar or submicromolar doses typically required to block Orai channels (Takahashi et al. 2014; Lee et al. 2017), and is believed to be primarily mediated by TRPC channels (Heo et al. 2012) in a Gd^3+^-insensitive manner (DeHaven et al. 2009). Our findings clearly show that SOCE in HSG cells operates in a Gd^3+^-insensitive manner (Fig. 4). HSG cells are known to express TRPC channels, including TRPC1 and TRPC3, which contribute to SOCE and are involved in fluid secretion (Liu et al. 2000, 2007). The diversity in the combinations of SOCE components, such as the Orai subtype and STIM family members, allows for variations in signaling properties between cell types. Further studies on the SOCE characteristics in salivary gland cells could deepen our understanding of the mechanisms underlying hyposalivation and its multiple causes.

Our findings highlight Ca^2+^-mobilizing GPCRs, including M3 and H1 receptors and Orai-independent SOCE, as inhibitory targets of CPZ. Identifying the causal target of drug-induced hyposalivation could inform mitigation strategies. For CPZ-mediated hyposalivation, we propose that ZnR/GPR39 may be a promising candidate. Our previous study demonstrated the role of ZnR/GPR39 in aquaporin-5 translocation in salivary glands (Kim et al. 2019), a finding later confirmed in knockout mice (Melamed et al. 2025). While ZnR/GPR39 may be partially affected by CPZ, its stimulation—such as with Zn^2+^-containing mouthwash—could potentially counteract CPZ-induced hyposalivation, though further investigation is required. Nonetheless, we emphasize that identifying specific salivary gland targets of hyposalivation-inducing drugs is particularly valuable, as it may inform viable strategies for mitigating their adverse effects. Furthermore, our study holds experimental significance as it introduces an animal research model for investigating hyposalivation. Systematic studies examining drug-induced hyposalivation in animal models, including mice, remain relatively scarce. If animal models are needed to examine the secondary effects of hyposalivation, we propose that administering 30 µM CPZ could provide a controllable, reversible, and reliably reproducible mouse model for studying hyposalivation.

CPZ is known to act on various molecular targets and is associated with a number of side effects. However, these diverse action targets enable a wide range of drug repositioning that could expand CPZ’s therapeutic applications in the future. Recently, the potential use of CPZ in treating COVID-19 has been suggested (Nobile et al. 2020; Plaze et al. 2020; Stip et al. 2020; Fred et al. 2021). Clinical observations of a lower incidence of COVID-19 infection in psychiatric patients treated with CPZ led to the proposal of repurposing this drug for non-psychiatric patients with COVID-19 (Plaze et al. 2020). Although the exact mechanism underlying CPZ’s antiviral activity remains unclear, it has been hypothesized to involve the inhibition of clathrin-mediated endocytosis and an increase in intravesicular pH within lysosomes (Glebov 2020). For drug therapies to be more effective, it is crucial to preserve the desired therapeutic effects (e.g., D2 receptor modulation in the prefrontal cortex, or antiviral activity against SARS-CoV-2) while minimizing off-target effects (e.g., hyposalivation). More sophisticated control of side effects based on a deeper understanding of molecular mechanisms could achieve this balance. Therefore, understanding the mechanisms underlying CPZ’s side effects, such as hyposalivation, and developing strategies to mitigate them will further enhance the therapeutic potential of CPZ, including its advantages for drug repositioning.

Although CPZ has been widely used, atypical antipsychotics, including clozapine, have demonstrated greater efficacy and are prescribed more frequently. A key limitation of this study is the inability to compare CPZ with atypical antipsychotics in terms of their effects on salivation mechanisms. Notably, clozapine induces sialorrhea (i.e. hypersalivation) rather than hyposalivation (Praharaj et al. 2006), suggesting totally distinct mechanisms of action in the salivary glands compared to their similar effects on the central nervous system. Further research into the regulation of salivary gland function by atypical antipsychotics may yield valuable insights.

## Supplementary Information

Below is the link to the electronic supplementary material.

Supplementary file1 (DOCX 598 KB)

## Data Availability

All source data for this work (or generated in this study) are available upon reasonable request.
