# Supplementary material for: Chlorpromazine induces hyposalivation by inhibiting muscarinic Ca2+ signaling in salivary glands
Source: Naunyn Schmiedebergs Arch Pharmacol. 2025 Jul 23;399(1):941–51. doi: 10.1007/s00210-025-04438-8 (PMC12894136; doi:10.1007/s00210-025-04438-8)
Supplement: Supplementary file 1 — Supplementary file1 (DOCX 598 KB) [file 210_2025_4438_MOESM1_ESM.docx]

**Supplementary Information**

**Chlorpromazine Induces Hyposalivation by Inhibiting Muscarinic Ca^2+^ Signaling in Salivary Glands**

Yoon-Jung Kim^1,^*, Yoobin Kim^1,^*, Soohyun Kim^1^, Tae-Yong Choi^1^, Hee-Kyung Park^2,3^, and Se-Young Choi^1^

^1^Department of Physiology, Dental Research Institute, Seoul National University School of Dentistry, Seoul 03080, Republic of Korea

^2^Department of Oral Medicine and Oral Diagnosis, Dental Research Institute, Seoul National University School of Dentistry, Seoul 03080, Republic of Korea.

^3^Department of Oral Medicine, Seoul National University Dental Hospital, Seoul 03080, Republic of Korea.

This Supplementary Information includes

Figure S1.

Figure S2.

Figure S3.


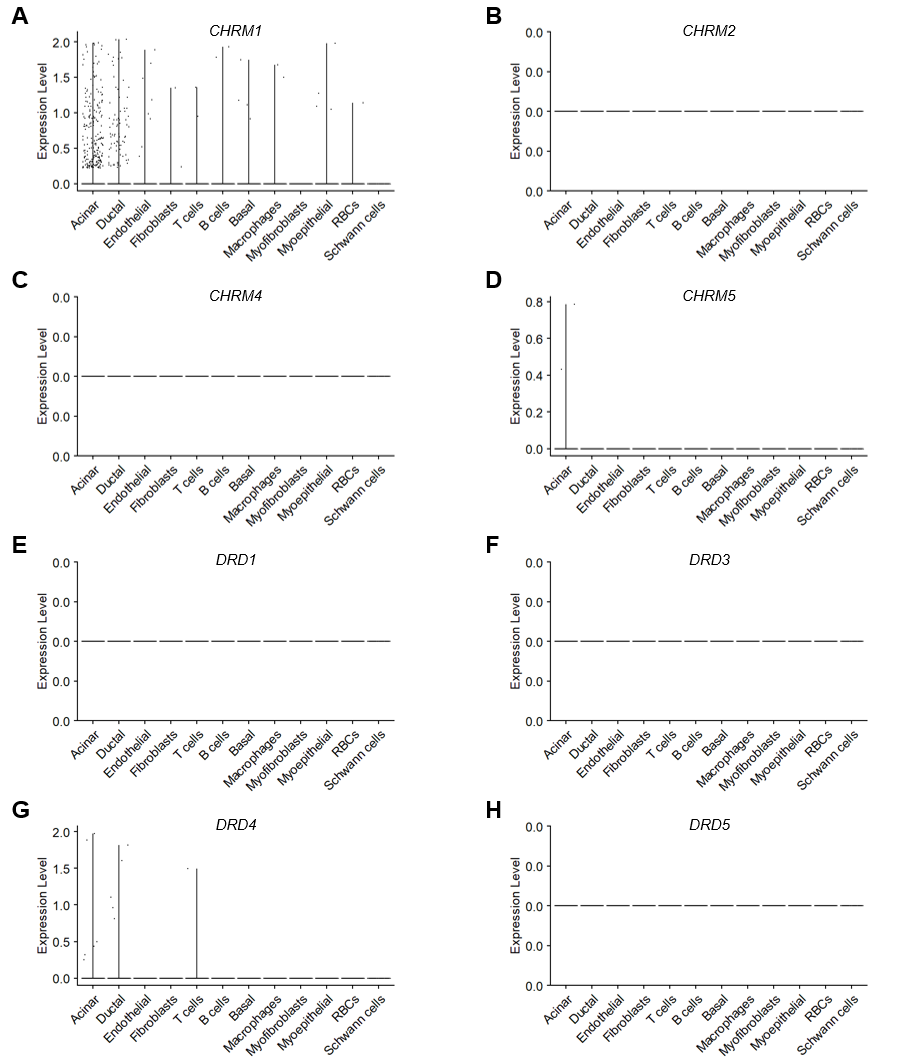


**Figure S1. mRNA expression of muscarinic and dopamine receptors in human SMGs, analyzed by scRNA-seq database.** (A-D) Violin plots showing expression of muscarinic receptors, including *CHRM1* (A)*, CHRM2* (B)*, CHRM4* (C)*,* and *CHRM5* (D) genes in human SMGs. (E-H) Violin plots showing expression of dopamine receptors, including *DRD1* (E)*, DRD3* (F)*, DRD4* (G)*,* and *DRD5* (H) genes in human SMGs.


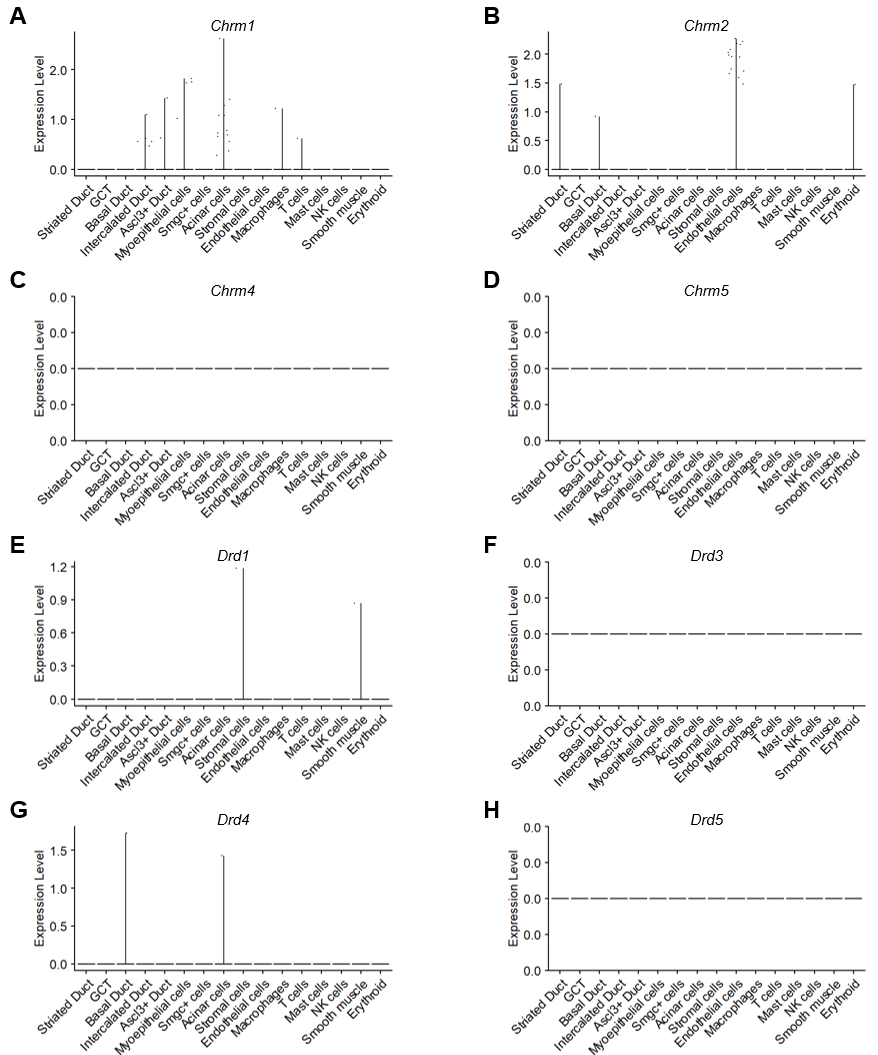


**Figure S2. mRNA expression of muscarinic and dopamine receptors in mouse SMGs, analyzed by scRNA-seq database**. (A-D) Violin plots showing expression of muscarinic receptors, including *Chrm1* (A), *Chrm2* (B)*, Chrm4* (C)*,* and *Chrm5* (D) genes in mouse SMGs. (E-H) Violin plots showing expression of dopamine receptors, including *Drd1* (E), *Drd3* (F), *Drd4* (G), and *Drd5* (H) genes in mouse SMGs.


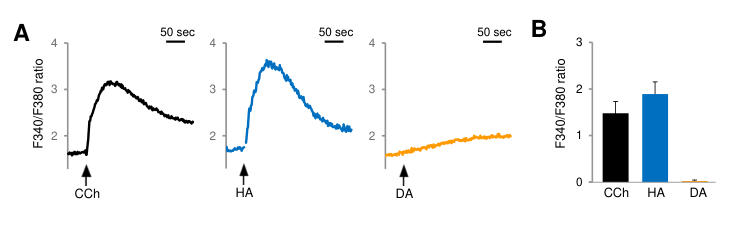


**Figure S3. Dopamine (DA) does not increase [Ca^2+^]_i_** **in salivary gland cells.** (A) Fura-2–loaded HSG cells were pretreated with 300 µM carbachol (CCh, black, left), 100 µM histamine (HA, blue, middle), and 500 µM dopamine (DA, orange, right). (B) The fluorescence ratio (F340/F380) was quantified to assess changes in [Ca^2+^]_i_ levels.
